# Betel quid use is associated with anemia among both men and women in Matlab, Bangladesh

**DOI:** 10.1371/journal.pgph.0001677

**Published:** 2023-06-14

**Authors:** Kristin K. Sznajder, Mary K. Shenk, Nurul Alam, Rubhana Raqib, Anjan Kumar, Farjana Haque, Tami Blumenfield, Siobhán M. Mattison, Katherine Wander

**Affiliations:** 1 Department of Public Health Sciences, Pennsylvania State University, College of Medicine, Hershey, Pennsylvania, United States of America; 2 Department of Anthropology, Pennsylvania State University, University Park, Pennsylvania, United States of America; 3 icddr,b, Dhaka, Bangladesh; 4 Department of Anthropology, University of New Mexico, Albuquerque, New Mexico, United States of America; 5 Department of Anthropology, Binghamton University, State University of New York, Binghamton, New York, United States of America; Sciensano, BELGIUM

## Abstract

Anemia accounts for 8.8% of total disability burden worldwide. Betel quid use among pregnant women has been found to increase anemia risk. Betel quid is prepared by wrapping the betel (or areca) nut, with spices and other additions, in betel or tobacco leaf and it is chewed or placed in the mouth. We explored the association between betel quid use and anemia among men and non-pregnant women. We collected data from a random sample of women and their husbands in Matlab, Bangladesh. Participants reported their current betel quid use and individual characteristics. We assessed hemoglobin (a biomarker of anemia) with a hemoglobinometer and soluble transferrin receptor (a biomarker of iron deficiency) and C-reactive protein (a biomarker of inflammation) in dried blood spots via enzyme immunoassay. We estimated logistic regression models to evaluate the association between betel quid use and anemia and structural equation models (SEM) to evaluate mediating roles of iron deficiency and elevated inflammation. A total of 1133 participants (390 men and 743 non-pregnant women) were included. After controlling for important confounders, any betel quid use was positively associated with anemia among men (OR: 1.80; 95% CI: 1.12, 2.89). Among women, betel quid use was associated with anemia only among the most frequent users (OR: 1.62; 95% CI: 1.03, 2.53). SEM did not reveal indirect paths through inflammation or iron deficiency. Betel quid use may contribute to the burden of anemia among adults in Bangladesh. Our findings suggest the burden of disease attributed to betel quid use has been underestimated.

## Introduction

Betel (or areca) nut is the seed of the areca palm (*areca catechu*) and an addictive stimulant used by 10% of the world’s population, primarily in South and Southeast Asia, East Africa, and the Pacific [1, 2]. Betel preparations vary by region, but generally involve a quid containing a betel nut wrapped in either a betel or tobacco leaf, which is then placed in the mouth or chewed dry or fresh [3, 4]. Reported motivations for betel quid use include improving the smell of the breath, reducing nausea, removing or preventing parasites, satisfying hunger, promoting digestion, or mitigating symptoms of poor mental health [5–7].

Betel quid consumption has extensive physiological effects across multiple organ systems, including the nervous, cardiovascular, gastrointestinal, respiratory, endocrine, and reproductive systems [1]. It is associated with preterm birth and low birth weight when used during pregnancy [1, 8]. Studies suggest betel quid use is associated with esophageal, liver, and oral cancers, diabetes, hypertension, and cardiovascular disease [1]. With the exception of esophageal and liver cancers, it is not clear that this effect is attributable to betel nut itself, or to other ingredients, such as tobacco, included in many betel quid preparations [9, 10]. While research has found an association between betel quid use and both anemia and folate deficiency among pregnant women [11–13], whether betel quid use is associated with anemia among the broader population remains an open question.

The global prevalence of anemia is 32.9% and anemia accounts for 8.8% of the total worldwide disability burden [14]. The prevalence of anemia in Bangladesh is substantially higher [15–17]. Anemia contributes to declines in physical and cognitive functioning in elderly populations, and reduces productivity among working age adults [14]. Anemia among the working age adult population contributes to reduced labor productivity, continuing cycles of poverty [18].

Betel quid use may cause anemia through multiple pathways, including blood loss, inflammation, and iron deficiency. Although animal studies suggest areca extract may have anti-inflammatory effects [19], oral use and the quid preparation likely cause tissue damage and trauma in the mouth and gastrointestinal tract, which may result in inflammation and/or blood loss [20–24], both of which can contribute to anemia [22]. Further, if betel quid use modulates immunity in a way that increases risk for infection, this may contribute to anemia [23, 24]. Betel quid’s appetite-suppressing effects [25, 26] may also reduce food intake, potentially causing anemia via deficiency in iron, folate, and other micronutrients [13].

Public health experts have called for increased research on the health effects of betel quid use [27]. Here, we contribute to this body of research by investigating associations between betel quid use and anemia among men and non-pregnant women, controlling for important socioeconomic confounders. In addition, we assessed two potential causal pathways, inflammation and iron deficiency.

## Methods

### Research setting

Data were collected in rural Matlab, Bangladesh. The region is undergoing rapid economic development and an epidemiological transition in which infectious disease rates have fallen while chronic diseases have become more prevalent, and rates of both undernutrition and obesity are high [28–30]. Betel quid is commonly used by both men and women in Matlab. While betel quid is widely consumed in small quantities at festive events, daily use of larger quantities is perceived in our study population (and documented among our study participants) to be more common among those with less education (p<0.001) and in older age groups (p<0.001).

### Study design and participants

Participants were originally recruited in 2010 by Shenk and Alam, when 944 women were randomly selected from a population roster housed by icddr,b (formerly known as the International Centre for Diarrhoeal Disease Research, Bangladesh) and were eligible to participate if they were between 20 and 65 years of age (equal numbers were drawn from women 20–34, 35–49, and 50–65). The data reported in this manuscript are from the second study wave conducted in 2018, in which survey, anthropometric, and biomarker data were collected from 765 (98%) of living 2010 participants still resident in the study area, as well as 499 of their living and co-resident husbands (many families had outmigrated between waves, and labor migrant husbands were generally not available for interview) [31–33].

### Measurements

#### Surveys

Surveys were piloted by Shenk and Alam and implemented by trained field staff in Matlab. Surveys collected individual and household information, including the exposure variable of interest, betel quid use, and important sociodemographic confounders including: participant’s year of birth, education (years), the MacArthur Scale of Subjective Social Status (MacArthur Ladder, a measure of socioeconomic status) [34, 35], food security (always or not always having enough quality food), food source (all compared with some or no food regularly procured from the bazaar), as well as use of tobacco and secondhand exposure to tobacco smoke at home.

#### Anthropometry and specimen collection

A small amount of capillary blood was collected via finger stick. Hemoglobin (Hb) concentration was immediately estimated with a hemoglobinometer (HemoCue 201+). Up to five additional drops of blood were allowed to fall onto filter paper (Whatman #903) for dried blood spots (DBS). DBS were allowed to dry at ambient temperature for up to 24 hours and then stored frozen at -20° C until laboratory evaluation. Height (cm) was estimated with a portable stadiometer (Seca 213) and weight (kg) was estimated with a portable digital scale (Tanita BC 545) [36].

#### Laboratory analyses

Concentrations of soluble transferrin receptor (sTfR), a biomarker of iron deficiency, and C-reactive protein (CRP), a biomarker of inflammation, were estimated by enzyme immunoassay (Ramco TFC-94 for sTfR and BioCheck BC-1119 for CRP), modified for use with DBS, in the icddr,b Immunobiology, Nutrition, and Toxicology Laboratory. One 3.2 mm disc of DBS specimen, equivalent to 1.5 μl of serum, was removed with a manual punch and eluted overnight in assay buffer at 4° C; eluent was then assayed without further dilution according to kit instructions. On 37 CRP plates evaluated for this project, the intra-assay coefficient of variation was 5.7% and the inter-assay coefficient of variation was 16.3% (16.7% for a second control specimen included on 27 of these plates). On 35 sTfR plates evaluated for this project, the intra-assay coefficient of variation (CV) was 3.5%, and the inter-assay CV was 14.8% at low concentration and 9.5% at high concentration [37–39]. Anemia was defined as Hb < 12 g/dl for women and < 13 g/dl for men as recommended by the World Health Organization [40]. Iron deficiency was defined as sTfR ≥ 5mg/l as this cut off has been shown to have the best combination of sensitivity and specificity for iron deficiency for this assay and method [41]. By convention, CRP ≥ 10 mg/l are typically excluded from analyses of chronic disease outcomes, to avoid conflating transient elevations due to acute infectious diseases with chronic background inflammation [42, 43]; following this convention, elevated inflammation was defined as 3 mg/l < CRP < 10 mg/l. Body mass index (BMI) was calculated as weight (in kg) divided by height (in m) squared, and underweight was defined as BMI < 18.5 kg/m [44].

### Statistical analysis

The outcome of interest was anemia and the main exposure of interest was current betel quid use. Betel quid use was assessed through a dichotomous variable for current use (yes, no) and also categorized by frequency of reported use: not a current user, infrequent user (at least once monthly but less than daily), low daily user (less than five times per day), or high daily user (at least five times per day), to examine effects of heavy use and the dose-response nature of any associations.

Adjustment variables were age, education, MacArthur Ladder, food security, food source, tobacco use, and exposure to secondhand tobacco smoke. We used both MacArthur Ladder and education to adjust for socioeconomic status [45, 46]. We avoided use of current income, because it could substantially underestimate the wealth of older participants who relied primarily on support from adult children, rather than their own income. We also examined household asset score as a control variable instead of MacArthur Ladder, which did not change our findings and did not improve the model fit.

We used logistic regression to estimate crude and adjusted odds ratios. We also estimated logistic regression models with potential mediator variables (elevated inflammation and iron deficiency). Finally, we used structural equation modeling (SEM) to estimate path coefficients to describe the effects of mediator variables: elevated inflammation and iron deficiency. All data were analyzed in SAS 9.4.

### Research ethics approval

The study was approved by the Ethical Review Committee at icddr,b and the Pennsylvania State University Institutional Review Board. Participants were informed the study was voluntary and provided written consent to participate; they were also provided with their anemia status, height, weight, BMI, and other health information at the time of data collection.

## Results

Anemia data were available for 1133 participants (390 men and 743 women; Table 1). A greater proportion of women were anemic (48.6%) compared with men (40.3%). Betel quid was used by 51.8% of men and 53.3% of women; more men (33.1%) than women (21.2%) reported the highest category of betel quid use (at least five times a day) (p<0.001). Men were older (p<0.001), had more years of education (p = 0.009), smoked tobacco at higher rates (p<0.001), and were more likely to be underweight (p<0.001) than women. Women reported higher scores on MacArthur Ladder (p<0.001), more often obtained all of their food from the bazaar (p<0.001), and were exposed to passive smoke at higher rates (p<0.001) than men.

**Table 1:** Characteristics of the study population.

	Total n = 1133 N (%)/ Mean (SD)	Men n = 390 N (%)/ Mean (SD)	Women n = 743 N (%)/ Mean (SD)	p-value
Betel quid use	598 (52.8)	202 (51.8)	396 (53.3)	0.630
No betel quid use	535 (47.2)	188 (48.2)	347 (46.7)	
Betel quid dose				
Never	535 (47.7)	188 (48.2)	347 (47.4)	<0.001
Infrequent	45 (4.0)	8 (2.1)	37 (5.1)	
Low Daily	258 (22.9)	65 (16.7)	193 (26.4)	
High Daily	284 (25.3)	129 (33.1)	155 (21.2)	
Age (continuous)	51.9 (12.6)	55.9 (12.1)	49.9 (12.3)	<0.001
MacArthur Ladder (continuous)	4.3 (1.7)	3.9 (1.2)	4.6 (1.9)	<0.001
Education (continuous)	4.3 (4.1)	4.7 (4.3)	4.0 (3.9)	0.009
Food secure	754 (66.8)	261 (67.1)	493 (66.6)	0.873
Not food secure	375 (33.2)	128 (32.9)	247 (33.4)	
All food from the bazaar	660 (58.5)	188 (48.7)	472 (63.6)	<0.001
Not all food from bazaar	469 (41.5)	199 (51.4)	270 (36.4)	
Elevated inflammation (CRP >3-10)	240 (22.0)	70 (18.7)	170 (23.7)	0.055
Low inflammation (CRP < = 3)	851 (78.0)	305 (81.3)	546 (76.3)	
Anemia	518 (45.7)	157 (40.3)	361 (48.6)	0.008
No anemia	615 (54.3)	233 (59.7)	382 (51.4)	
Iron deficiency (sTfR >5 mg/l)	570 (50.4)	180 (46.2)	390 (52.6)	0.038
No iron deficiency (sTfR = < 5 mg/l)	561 (49.6)	210 (53.9)	351 (47.4)	
Total children	3.7 (2.0)	N/A	3.7 (2.0)	N/A
Smoke	156 (13.8)	156 (40.0)	0 (0.0)	<0.001
Does not smoke	977 (86.2)	234 (60.0)	743 (100.0)	
Secondhand smoke	287 (25.3)	22 (5.6)	265 (35.7)	<0.001
No secondhand smoke	846 (74.7)	368 (94.4)	478 (64.3)	
Underweight	160 (14.2)	85 (22.0)	75 (10.1)	<0.001
Not underweight	968 (85.8)	302 (78.0)	666 (89.9)	
BMI (continuous)	22.9 (4.2)	21.5 (3.7)	23.6 (4.3)	<0.001

Gender interacted with betel quid use in logistic regression models of anemia after controlling for confounders common for both men and women (coefficient for betel quid use*gender: 0.634; p: 0.019), therefore subsequent modeling was stratified by gender. We assessed interactions between betel quid use and age for men and women and did not identify any.

A crude positive association between betel quid use and anemia was apparent among men (crude odds ratio (cOR): 2.58; 95% CI: 1.69, 3.93; Table 2). Among men (Table 2, Panel 2), betel quid use remained independently positively associated with anemia (adjusted odds ratio (aOR): 1.80; 95% CI: 1.12, 2.89) after controlling for confounders. The covariates also associated with anemia among men were age (aOR: 1.04; 95% CI: 1.02, 1.06), education (aOR: 0.94; 95% CI: 0.89, 0.99), and smoking (aOR: 0.54; 95% CI: 0.34, 0.87). When potential mediators (elevated inflammation and iron deficiency) were included in the multiple regression model for men (Table 2, Panel 3), anemia remained independently associated with betel quid use (aOR: 1.84; 95% CI: 1.12, 3.03), as did age (aOR: 1.05; 95% CI: 1.02, 1.07), education (aOR: 0.93; 95% CI: 0.88, 0.99), and smoking (aOR: 0.55; 95% CI: 0.34, 0.90). Neither inflammation nor iron deficiency were associated with anemia.

**Table 2:** Logistic regression models of anemia (hemoglobin<13) among men n = 390.

**Panel 1**	**OR (CI) (n = 390)**
Betel quid use	2.58 (1.69, 3.93)
**Panel 2**	**OR (CI) (n = 381)**
Betel quid use	1.80 (1.12, 2.89)
Age	1.04 (1.02, 1.06)
MacArthur Ladder	0.99 (0.81, 1.23)
Education	0.94 (0.89, 0.99)
Smokes	0.54 (0.34, 0.87)
Food secure	0.88 (0.53, 1.47)
All food from the bazaar	0.87 (0.55, 1.39)
**Panel 3**	**OR (CI) (n = 366)**
Betel quid use	1.84 (1.12, 3.03)
Age	1.05 (1.02, 1.07)
MacArthur Ladder	1.02 (0.81, 1.27)
Education	0.93 (0.88, 0.99)
Smokes	0.55 (0.34, 0.90)
Food secure	0.89 (0.52, 1.52)
All food from the bazaar	0.84 (0.52, 1.37)
Elevated inflammation	1.76 (0.98, 3.16)
Iron deficiency	1.25 (0.79, 1.99)

Because only eight men reported infrequent use of betel quid, we combined this category with daily low use in regression models. When betel quid use was categorized by level of use (none/infrequent + daily low (less than 5 times per day)/daily high (at least 5 times per day)), shown in Table 3, Panel 2, infrequent + daily low and daily high betel quid use were associated with anemia compared with no betel quid use (aOR: 2.94; 95% CI: 1.52, 5.67 and aOR: 1.69; 95% CI: 0.99, 2.88 respectively). A test for trend supported a dose-response relationship, although it was not statistically significant at the 0.05 alpha level (aOR: 1.29; 95% CI: 0.99, 1.69).

**Table 3:** Logistic regression models of anemia (hemoglobin<13) among men n = 390.

**Panel 1**	**OR (CI) (n = 390)**
Betel quid use	
None	REF
Infrequent and Low Daily	3.70 (2.06, 6.64)
High Daily	2.67 (1.68, 4.26)
**Panel 2**	**OR (CI) (n = 381)**
Betel quid use	
None	REF
Infrequent and Low Daily	2.94 (1.52, 5.67)
High Daily	1.69 (0.99, 2.88)
Age	1.04 (1.02, 1.06)
MacArthur Ladder	1.01 (0.82, 1.25)
Education	0.94 (0.89, 0.99)
Smokes	0.49 (0.30, 0.79)
Food Secure	0.87 (0.52, 1.46)
All Food from the Bazaar	0.89 (0.56, 1.41)
**Panel 3**	**OR (CI) (n = 366)**
Betel quid use	
None	REF
Infrequent and Low Daily	3.34 (1.48, 6.66)
High Daily	1.65 (0.95, 2.88)
Age	1.05 (1.02, 1.07)
MacArthur Ladder	1.03 (0.83, 1.29)
Education	0.93 (0.88, 0.99)
Smokes	0.47 (0.28, 0.79)
Food Secure	0.88 (0.51, 1.49)
All Food from the Bazaar	0.86 (0.53, 1.39)
Elevated Inflammation	1.72 (0.94, 3.12)
Iron Deficiency	1.19 (0.74, 1.90)

A crude positive association between betel quid use and anemia was apparent among women (cOR: 1.63; 95% CI: 1.22, 2.19; Table 4). Among women, betel quid use was not associated with anemia when confounders were added to the model (aOR: 1.36; 95% CI: 0.94, 1.97) (Table 4, Panel 2). Age was positively associated with anemia (aOR: 1.03; 95% CI: 1.01, 1.05), while MacArthur Ladder (aOR: 0.89; 95% CI: 0.81, 0.97), and secondhand smoke exposure (aOR: 0.69; 95% CI: 0.51, 0.95) were inversely associated with anemia. These patterns remained largely unchanged by inclusion of potential mediators (Table 4, Panel 3), and possible mediators were not associated with anemia (elevated inflammation: aOR: 0.86; 95% CI: 0.59, 1.24 and iron deficiency: aOR: 1.04; 95% CI: 0.77, 1.41). When betel quid use was considered as a categorical variable (Table 5, Panel 2), the highest category of use was associated with anemia compared with no betel quid use (aOR: 1.62; 95% CI: 1.03, 2.53), but less frequent use categories were not associated with anemia. A test for trend supported a dose-response relationship (aOR: 1.17; 95% CI: 1.01, 1.36).

**Table 4:** Logistic regression models of anemia (hemoglobin<12) among women n = 743.

**Panel 1**	**OR (CI) (n = 743)**
Betel quid use	1.63 (1.22, 2.19)
**Panel 2**	**OR (CI) (n = 732)**
Betel quid use	1.36 (0.94, 1.97)
Age	1.03 (1.01, 1.05)
MacArthur Ladder	0.89 (0.81, 0.97)
Education	1.04 (0.99, 1.09)
Total children	0.99 (0.91, 1.09)
Secondhand smoke	0.69 (0.51, 0.95)
Food Secure	0.98 (0.69, 1.38)
All Food from the Bazaar	0.91 (0.67, 1.25)
**Panel 3**	**OR (CI) (n = 705)**
Betel quid use	1.32 (0.91, 1.93)
Age	1.03 (1.01, 1.05)
MacArthur Ladder	0.89 (0.81, 0.97)
Education	1.04 (0.99, 1.09)
Total children	0.99 (0.91, 1.09)
Secondhand smoke	0.73 (0.52, 1.01)
Food Secure	1.00 (0.71, 1.43)
All Food from the Bazaar	0.89 (0.65, 1.24)
Elevated Inflammation	0.86 (0.59, 1.24)
Iron Deficiency	1.04 (0.77, 1.41)

**Table 5:** Logistic regression models of anemia (hemoglobin<12) among women n = 743.

**Panel 1**	**OR (CI) (n = 732)**
Betel quid use	
None	REF
Infrequent	1.05 (0.53, 2.08)
Low Daily	1.58 (1.11, 2.25)
High Daily	1.96 (1.33, 2.88)
**Panel 2**	**OR (CI) (n = 722)**
Betel quid use	
None	REF
Infrequent	0.89 (0.44, 1.82)
Low Daily	1.29 (0.84, 1.99)
High Daily	1.62 (1.03, 2.53)
Age	1.03 (1.01, 1.05)
MacArthur Ladder	0.88 (0.81, 0.97)
Education	1.04 (0.99, 1.09)
Total children	0.99 (0.91, 1.09)
Secondhand smoke	0.69 (0.51, 0.95)
Food Secure	1.01 (0.71, 1.42)
All Food from the Bazaar	0.93 (0.67, 1.27)
**Panel 3**	**OR (CI) (n = 695)**
Betel quid use	
None	REF
Infrequent	0.91 (0.44, 1.88)
Low Daily	1.19 (0.77, 1.86)
High Daily	1.59 (1.01, 2.53)
Age	1.03 (1.01, 1.05)
MacArthur Ladder	0.88 (0.81, 0.97)
Education	1.04 (0.98, 1.09)
Total children	0.99 (0.91, 1.09)
Secondhand smoke	0.73 (0.53, 1.02)
Food Secure	1.03 (0.73, 1.47)
All Food from the Bazaar	0.91 (0.66, 1.26)
Elevated Inflammation	0.81 (0.56, 1.18)
Iron Deficiency	1.09 (0.80, 1.49)

**Fig 1:**
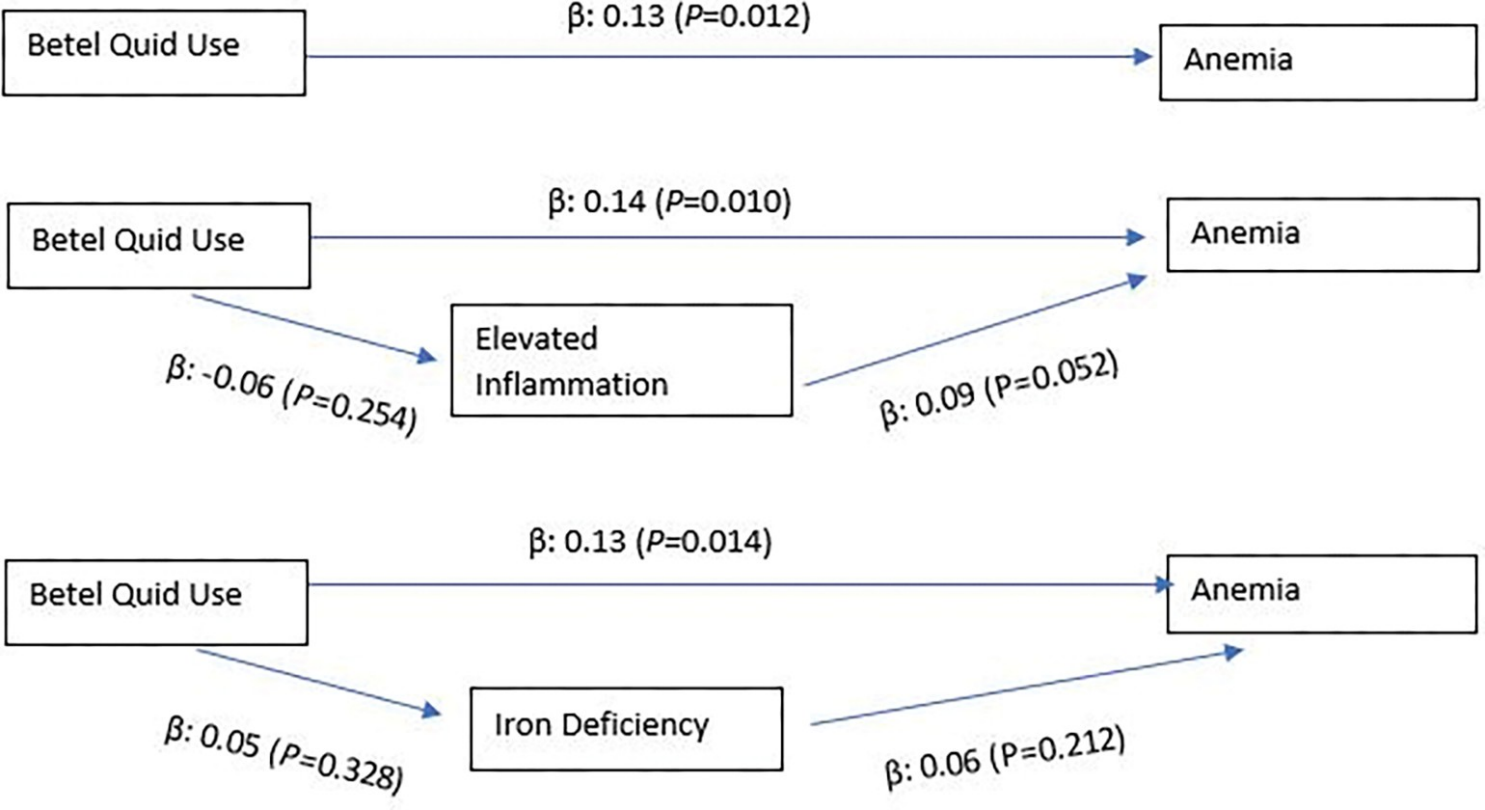
Structural equation models to assess the direct and indirect pathways in the association between betel quid use and anemia among men.

**Fig 2:**
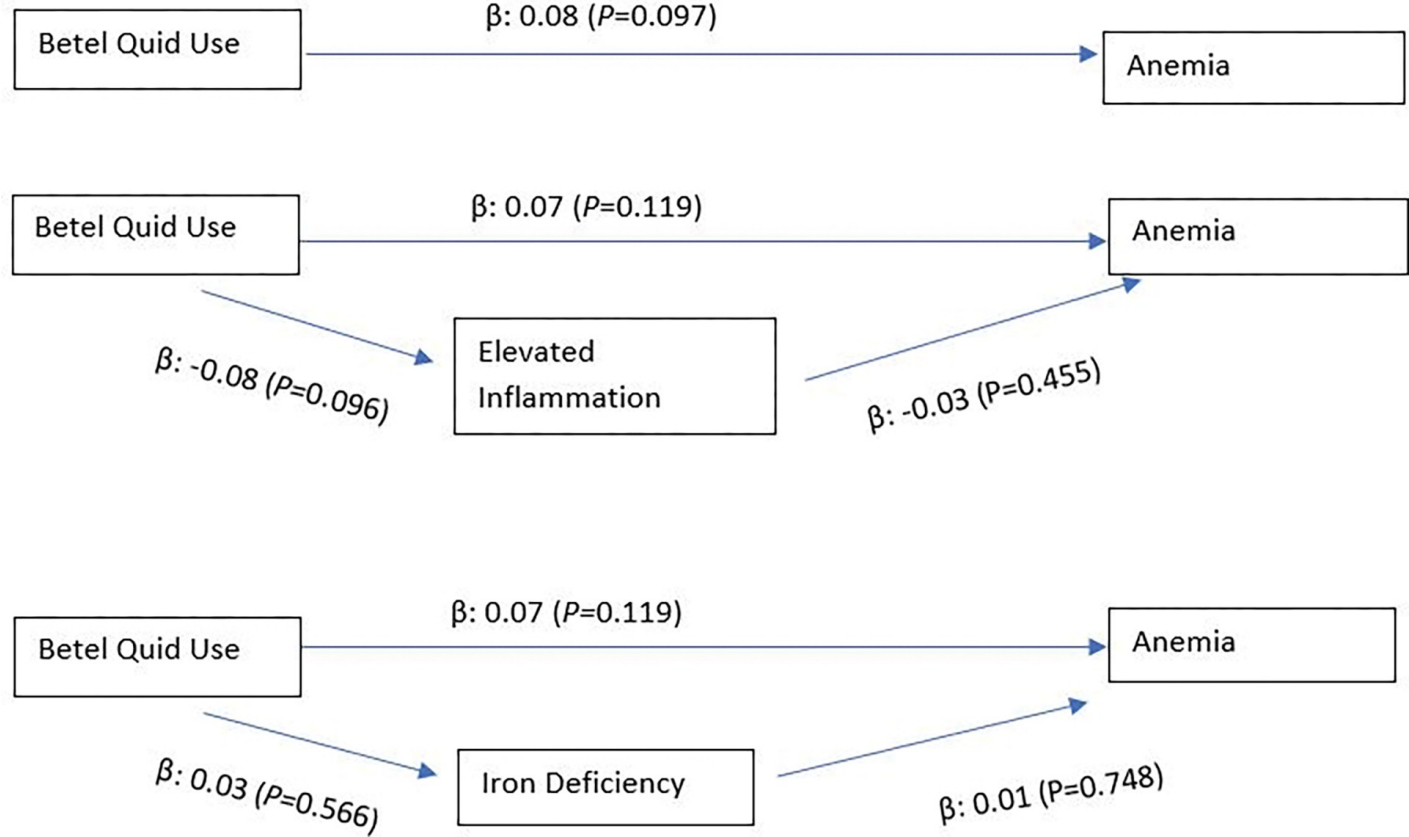
Structural equation models to assess the direct and indirect pathways in the association between betel quid use and anemia among women.

Among men, SEM showed a direct path between betel quid use and anemia (β: 0.13; p: 0.012), but no indirect paths were found through iron deficiency (betel quid use to iron deficiency: β: 0.05; p: 0.328; iron deficiency to anemia: β: 0.06; p: 0.212) or inflammation (betel quid use to inflammation: β: -0.06; p: 0.254; inflammation to anemia: β: 0.09; p: 0.052) (Fig 1). Among women, SEM did not reveal direct path between betel quid use and anemia (β: 0.08; p: 0.097) nor mediating paths through iron deficiency (betel quid use to iron deficiency: β: 0.03; p: 0.566; iron deficiency to anemia: β: 0.01; p: 0.748), nor through inflammation (betel quid use to inflammation: β: -0.08; p: 0.096; inflammation to anemia: β: -0.03; p: 0.455) (Fig 2). All SEM controlled for the same confounding variables in Panel 2 in Tables 2–5.

Underweight was independently positively associated with anemia among women (OR: 1.77; 95% CI: 1.05, 3.00), but not men (OR: 1.47; 95% CI: 0.81, 2.66), after controlling for confounders and mediators (S1 Table). However, because underweight is not itself a cause of anemia but instead a correlate of iron and other micronutrient deficiencies, the interpretability of these models is limited.

## Discussion

Betel quid use was associated with anemia among men and non-pregnant women, although the association in women was limited to the most frequent users. Tests for trend suggest a dose-response relationship (increasing frequency of betel quid use was associated with increased risk for anemia) among both men and women. These observations suggest betel quid use may increase anemia risk for both genders. When we investigated direct and indirect pathways, we found evidence for both direct effects, and, among women, a pathway through iron deficiency.

Our finding that betel quid use may contribute to anemia among men and non-pregnant women adds needed breadth to the current research regarding health effects of betel quid use, which has largely focused on betel quid’s association with adverse pregnancy outcomes as well as oropharyngeal and gastrointestinal cancers. Increasing risk for anemia may be one of betel quid’s most impactful effects on users’ health, as anemia reduces the ability to work and contributes to risk for other illnesses. The high prevalence of anemia in Bangladesh, and our findings showing direct and indirect pathways from betel quid use to anemia, suggest the disease burden attributable to betel quid use may be substantially higher than previously appreciated.

Multiple physiological pathways might explain the direct effects of betel quid use and anemia suggested by our findings. Betel quid use might cause irritation or blood loss in the mouth or gastrointestinal tract, which could lead to anemia [47, 48]. Using betel quid might also have direct physiological effects on hemoglobin production, erythropoiesis, or erythrocyte longevity, or adversely affect these via oxidative stress [49]. Betel quid use might also act through mediating variables not considered here, such as immune suppression and increased risk for infections that can cause anemia [50]. In addition, tannins or other components of betel quid may cause damage to tissues of the gastrointestinal tract leading to blood loss.

There are several important strengths to this analysis, including its large sample size, the evaluation of direct and indirect pathways through inflammation and iron deficiency, and the inclusion of important covariates. However, we note several important limitations. Our ability to make causal inference is limited by the cross-sectional (we cannot be certain betel quid use preceded anemia) and observational (participants were not randomized to receive betel quid) nature of the study design. Some of the associations we observed had wide confidence intervals and/or confidence intervals that included (or came close to including) the null effect; this may be due to small numbers (e.g., of women in the highest frequency category of betel quid use) and should be interpreted with caution. The survey data relied on self-report and may be vulnerable to bias (some participants may have been more likely than others to under-report betel quid use). Additionally, our analyses are limited by the potential for unmeasured confounding due to infectious diseases (e.g., hookworm), other components of the diet not collected through the survey, and contraceptive use. This study did not capture how participants typically prepared betel quid, therefore we cannot speak to how individual ingredients might contribute to anemia—it is possible a non-betel ingredient, like chewing tobacco, explains some or all of the association between betel quid use and anemia [13, 51, 52]. Furthermore, there may be pathways through inflammation or appetite suppression that we were not able to capture with biomarkers of inflammation and iron deficiency. It is also important to note the measurement of inflammation used in this study, CRP, is imperfect, and is not the only available biomarker of inflammation. Therefore, our data may have partially misclassified inflammation within our study population. The biomarker of iron deficiency we used, sTfR, while ideal in many ways for population-based research in remote settings, lacks consensus around a single definition of iron deficiency [53] and so may have misclassified some cases, potentially obscuring a pathway through iron deficiency. However, this kind of misclassification is expected to be non-differential, biasing results toward the null hypothesis, yet despite this we were clearly able to detect an association. Finally, the generalizability of our findings beyond rural Bangladesh remains to be evaluated.

## Conclusions

Our findings suggest betel quid use may increase risk for anemia. Current research underestimates the disease burden attributable to betel quid use particularly for men and non-pregnant women, among whom the burden of anemia remains substantial but chronically understudied, and for whom impairment or disability due to anemia is likely to have negative health and economic consequences for individuals and their families.

## Supporting information

S1 Table Logistic regression models of anemia including underweight. (DOCX)
