# Correction: Betel quid use is associated with anemia among both men and women in Matlab, Bangladesh

**DOI:** 10.1371/journal.pgph.0003444

**Published:** 2024-06-25

**Authors:** Kristin K. Sznajder, Mary K. Shenk, Nurul Alam, Rubhana Raqib, Anjan Kumar, Farjana Haque, Tami Blumenfield, Siobhán M. Mattison, Katherine Wander

There is an error in Table 3. The classification of eight men who used betel quid infrequently should be no betel quid used group not daily low group. Please see the correct Table 3 here.

**Table 3:** Logistic regression models of anemia (hemoglobin<13) among men n = 390.

**Panel 1**	**OR (CI) (n = 390)**
Betel quid use	
None or Infrequent	REF
Low Daily	3.70 (2.06, 6.64)
High Daily	2.67 (1.68, 4.26)
**Panel 2**	**OR (CI) (n = 381)**
Betel quid use	
None or Infrequent	REF
Low Daily	2.94 (1.52, 5.67)
High Daily	1.69 (0.99, 2.88)
Age	1.04 (1.02, 1.06)
MacArthur’s Ladder	1.01 (0.82, 1.25)
Education	0.94 (0.89, 0.99)
Smokes	0.49 (0.30, 0.79)
Food Secure	0.87 (0.52, 1.46)
All Food from the Bazaar	0.89 (0.56, 1.41)
**Panel 3**	**OR (CI) (n = 381)**
Betel quid use	
None or Infrequent	REF
Low Daily	3.34 (1.68, 6.66)
High Daily	1.65 (0.95, 2.88)
Age	1.05 (1.02, 1.07)
MacArthur’s Ladder	1.03 (0.83, 1.29)
Education	0.93 (0.88, 0.99)
Smokes	0.47 (0.28, 0.79)
Food Secure	0.88 (0.51, 1.49)
All Food from the Bazaar	0.86 (0.53, 1.39)
Elevated Inflammation	1.72 (0.94, 3.12)
Iron Deficiency	1.19 (0.74, 1.90)

In the fourth paragraph of Results, there is an error in the first two sentences. The correct sentence is: Because only eight men reported infrequent use of betel quid, we combined this category with no betel quid use in regression models. When betel quid use was categorized by level of use (none/infrequent daily low (less than 5 times per day), daily high (at least 5 times per day)), shown in Table 3, Panel 2, daily low and daily high betel quid use were associated with anemia compared with no/infrequent betel quid use (aOR: 2.94; 95% CI: 1.52, 5.67 and aOR: 1.69; 95% CI: 0.99, 2.88 respectively).
