# Supplementary material for: Betel quid use is associated with anemia among both men and women in Matlab, Bangladesh
Source: PLOS Glob Public Health. 2023 Jun 14;3(6):e0001677. doi: 10.1371/journal.pgph.0001677 (PMC10266624; doi:10.1371/journal.pgph.0001677)
Supplement: S1 Table — (DOCX) [file pgph.0001677.s001.docx]

| **S1 Table. Logistic regression models of anemia including underweight** | | |
| --- | --- | --- |
| **Variables** | **Men** | **Women** |
|  | **OR (CI) (n=364)** | **OR (CI) (n=703)** |
| Betel quid use | 1.85 (1.12, 3.06) | 1.32 (0.90, 1.93) |
| Age | 1.05 (1.03, 1.07) | 1.03 (1.01, 1.05) |
| MacArthur’s Ladder | 1.04 (0.83, 1.31) | 0.89 (0.82, 0.98) |
| Education | 0.93 (0.87, 0.99) | 1.04 (0.99, 1.09) |
| Total children | -- | 0.99 (0.91, 1.09) |
| Smokes | 0.52 (0.32, 0.86) | -- |
| Secondhand smoke | -- | 0.73 (0.53, 1.00) |
| Food secure | 0.90 (0.54, 1.46) | 1.04 (0.73, 1.47) |
| All food from the bazaar | 0.89 (0.54, 1.46) | 0.92 (0.66, 1.27) |
| Elevated inflammation | 1.85 (1.02, 3.38) | 0.89 (0.62, 1.29) |
| Iron deficiency | 1.29 (0.81, 2.09) | 1.06 (0.78, 1.44) |
| Underweight | 1.47 (0.81, 2.66) | 1.77 (1.05, 3.00) |
